# Anti-nuclear Matrix Protein 2 Antibody-Positive Dermatomyositis Relapse With Preceding Panniculitis

**DOI:** 10.7759/cureus.78622

**Published:** 2025-02-06

**Authors:** Hirokazu Taguchi, Yoshitaka Ueda, Yohya Shigehara, Naoto Yokogawa

**Affiliations:** 1 Department of Rheumatic Diseases, Tokyo Metropolitan Tama Medical Center, Fuchu, JPN; 2 Department of Dermatology, Tokyo Metropolitan Tama Medical Center, Fuchu, JPN

**Keywords:** anti-nuclear matrix protein 2 antibody, dermatomyositis, lipoatrophy, myositis-specific antibody, panniculitis, skin biopsy

## Abstract

Dermatomyositis (DM) is an idiopathic inflammatory myopathy characterized by cutaneous lesions such as heliotrope rash, Gottron's papules, and Gottron's sign. Panniculitis is rarely reported as a skin manifestation of DM. Herein, we present a case of anti-nuclear matrix protein 2 antibody (NXP-2)-positive DM relapse with panniculitis in the absence of muscle symptoms. A 32-year-old female patient was referred to our department. Nine years ago, she was diagnosed with DM and had received prednisolone (PSL) and tacrolimus therapy. However, she discontinued her visits four years ago. She most recently presented with erythematous lesions on the right thigh and buttocks without any muscle symptoms. Thirteen days later, she experienced upper limb weakness. Laboratory findings at her current presentation demonstrated elevated serum creatine kinase levels and positivity for anti-NXP-2 antibody. A skin biopsy of the erythema on her left thigh demonstrated findings consistent with healed panniculitis. Based on these findings, an anti-NXP-2 antibody-positive DM relapse was diagnosed. Her symptoms resolved with PSL and tacrolimus therapy. It is important to recognize that panniculitis is a significant, cutaneous manifestation of DM and can present as the initial manifestation of a DM relapse even in the absence of muscle symptoms.

## Introduction

Panniculitis is a rare manifestation of dermatomyositis (DM) [1]. In a systematic review of 91 cases of DM-panniculitis, the highest number of cases were reported from Asia, accounting for one-third of the total cases. [2]. It is not often reported as its initial manifestation or sole a cutaneous manifestation of DM [3,4]. Muscle symptoms typically accompany DM-associated panniculitis, and few cases in which panniculitis preceded muscle symptoms have been reported. The anti-nuclear matrix protein 2 (NXP-2) antibody is a common, myositis-specific antibody (MSA) in juvenile-onset DM (JDM) [5] and accounts for 1.6% to 25% of adult-onset DM cases as well [6]. The characteristics of anti-NXP-2-positive DM are severe muscle weakness, highly elevated serum creatinine kinase, dysphagia, subcutaneous edema, malignancy, and subcutaneous calcinosis. Panniculitis has rarely been reported in the context of anti-NXP-2 positive DM. Herein, we report a case of an anti-NXP-2-positive DM relapse with preceding panniculitis.

## Case presentation

**Figure 1:**
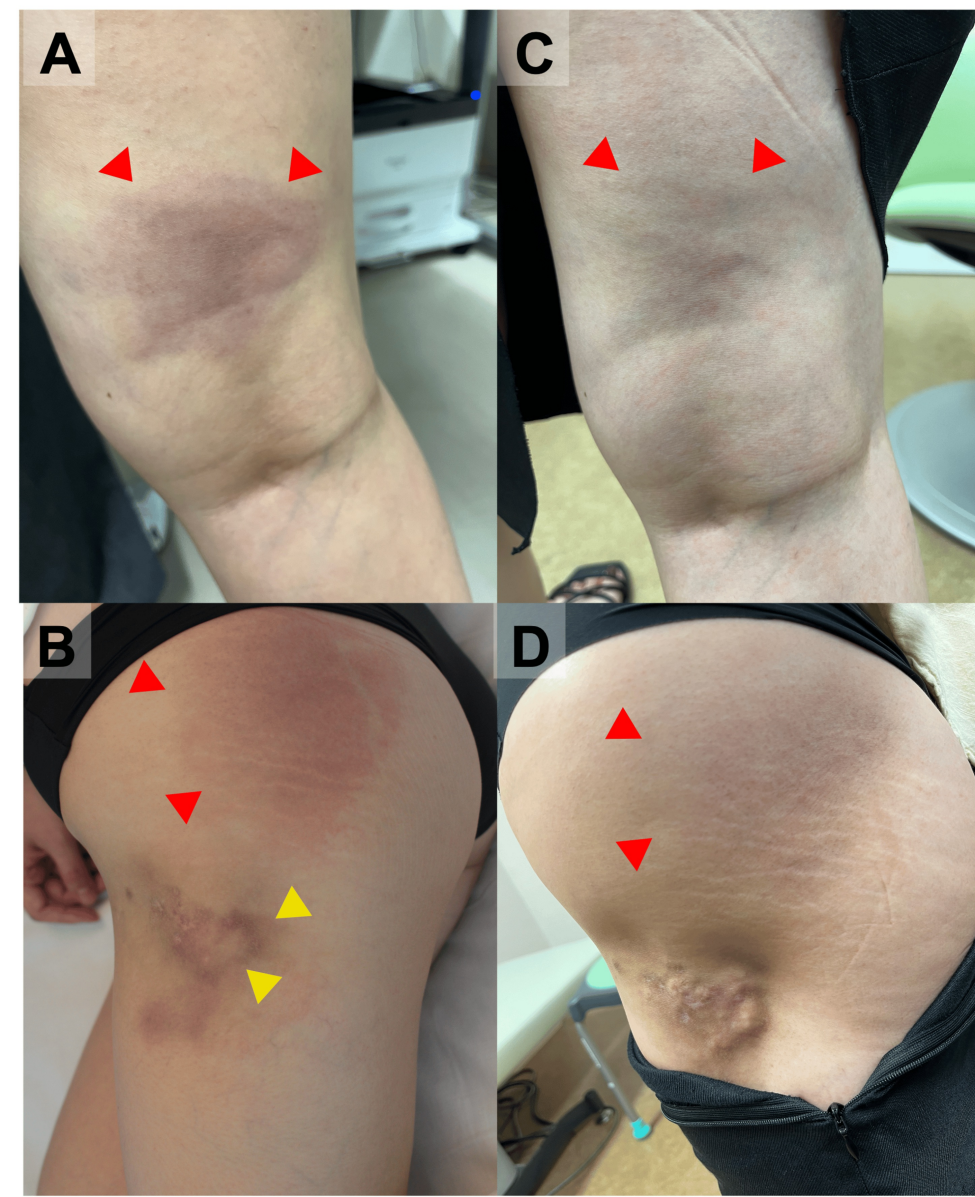
Cutaneous findings before (A, B) and after (C, D) treatment. (A, B) Erythema with tenderness on the right thigh and hip at the second visit (red arrows). Lipoatrophic skin lesion from nine years earlier on the left thigh (B, yellow arrows). (C, D) Erythema with tenderness on the right thigh and buttock following treatment.

A 32-year-old female patient was referred to our department for a one-month history of tender erythema of the right thigh and buttock. Nine years earlier, she had presented to our department with myalgia of the extremities. A physical examination at the time revealed a malar rash and tender erythema on the left thigh. Manual muscle testing (MMT) revealed the following muscle strength grades: deltoids: 3/3; biceps brachii: 4/4; iliopsoas: 3/3; quadriceps femoris: 3/3; and tibialis anterior: 5/5. Her serum CK level was 10,152 U/L (reference: 41-153 U/L). Tests for antinuclear antibody, anti-SS-A antibody, anti-DNA antibody, anti-Smith antibody, anti-ribonucleoprotein antibody, anti-aminoacyl tRNA synthetase (ARS) antibody, anti-melanoma differentiation-associated gene 5 (MDA5) antibody, anti-transcriptional intermediary factor 1 (TIF1)-γ antibody, and anti-Mi-2 antibody were negative. At the time, the anti-NXP-2 antibody was not assessed. Magnetic resonance imaging (MRI) of both thighs and electromyography of the left deltoid, which had been performed at the initial presentation nine years ago, demonstrated inflammatory myopathy. However, at the time, a muscle biopsy of the right vastus lateralis found no pathological evidence of inflammatory myopathy. DM was diagnosed and treated with prednisolone (PSL) 50 mg/day (1 mg/kg/day) and tacrolimus 2 mg/day. Subsequently, her muscle weakness and elevated CK improved. The erythema on the left thigh also improved, leaving a lipoatrophic area in the same region. No skin biopsy of the tender erythema was performed. Subsequently, the PSL, which was being administered concurrently with tacrolimus, was tapered to 4 mg/day without any relapse. Four years earlier, the patient had discontinued her hospital visits and immunosuppressive therapy. A physical examination at the current presentation revealed mildly palpable erythema with tenderness in the right thigh and hip (Figures 1A, 1B). The lipoatrophic area on the left thigh from nine years ago also remained (Figure 1B).

No facial rash or DM-specific rash, such as heliotrope rash, Gottron’s papules, or muscle weakness, was observed. Laboratory findings demonstrated normal CK and aldolase (ALD) (Table 1).

**Table 1:** Laboratory findings on current presentation WBC, White blood cell; ESR, Erythrocyte sedimentation rate; T-Bil; Total bilirubin; AST, aspartate aminotransferase; ALT, alanine aminotransferase; ALP, alkaline phosphatase; CK, creatine kinase; BUN, Blood urea nitrogen; Cre, Creatinine; CRP, C-reactive protein; C3, Complement 3; C4, Complement 4

Complete blood count		Biochemistry			
WBC	5.6 x 10^3^/μL	Albumin	4.1 g/dL	Na	139 mEq/L
Neutrophil	75%	T-Bil	0.7 mg/dL	K	3.9 mEq/L
Lymphocyte	20%	AST	22 U/L	CRP	0.25 mg/dL
Hemoglobin	13.6 g/dL	ALT	15 U/L	IgG	1,203 mg/dL
Platelet count	24.4 x 10^4^/μL	CK	149 U/L	C3	128 mg/dL
Hematological test		BUN	7.3 mg/dL	C4	17.7 mg/dL
ESR (1 hr)	6 mm	Cre	0.65 mg/dL	Aldolase	2.8 U/L

**Figure 2:**
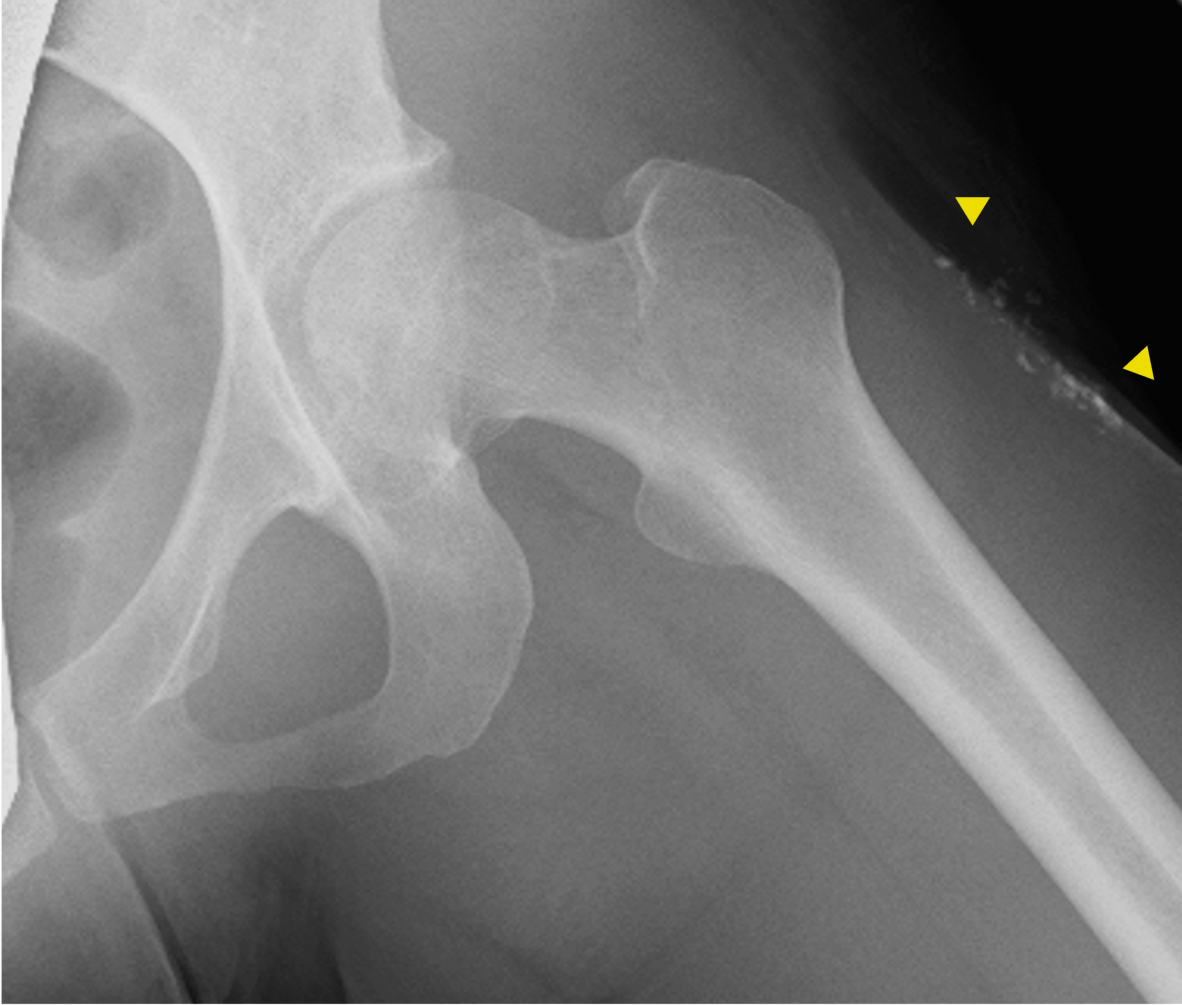
Radiograph of the left thigh showing subcutaneous calcinosis (arrows).

Tests for the ARS antibody, anti-MDA5 antibody, anti-TIF1-γ antibody, anti-Mi-2 antibody, anti-SRP antibody, and anti-HMGCR antibody returned negative. Thirteen days later, she returned to our department with newly developed upper limb weakness. A physical examination revealed new erythema on her left thigh, Gottron papules covering the dorsal aspect of the right interphalangeal joint, periungual erythema, and the holster sign. There was fatigue in the upper limbs. MMT revealed full muscle strength (5/5) in all the tested muscles. Laboratory findings demonstrated elevated levels of serum CK (1,010 U/L) and ALD (7.6 U/L; reference: 2.1-6.1 U/L). Anti-NXP-2 antibody detected by indirect immunofluorescence assay was positive. A radiograph of the left thigh demonstrated subcutaneous calcinosis (Figure 2).

**Figure 3:**
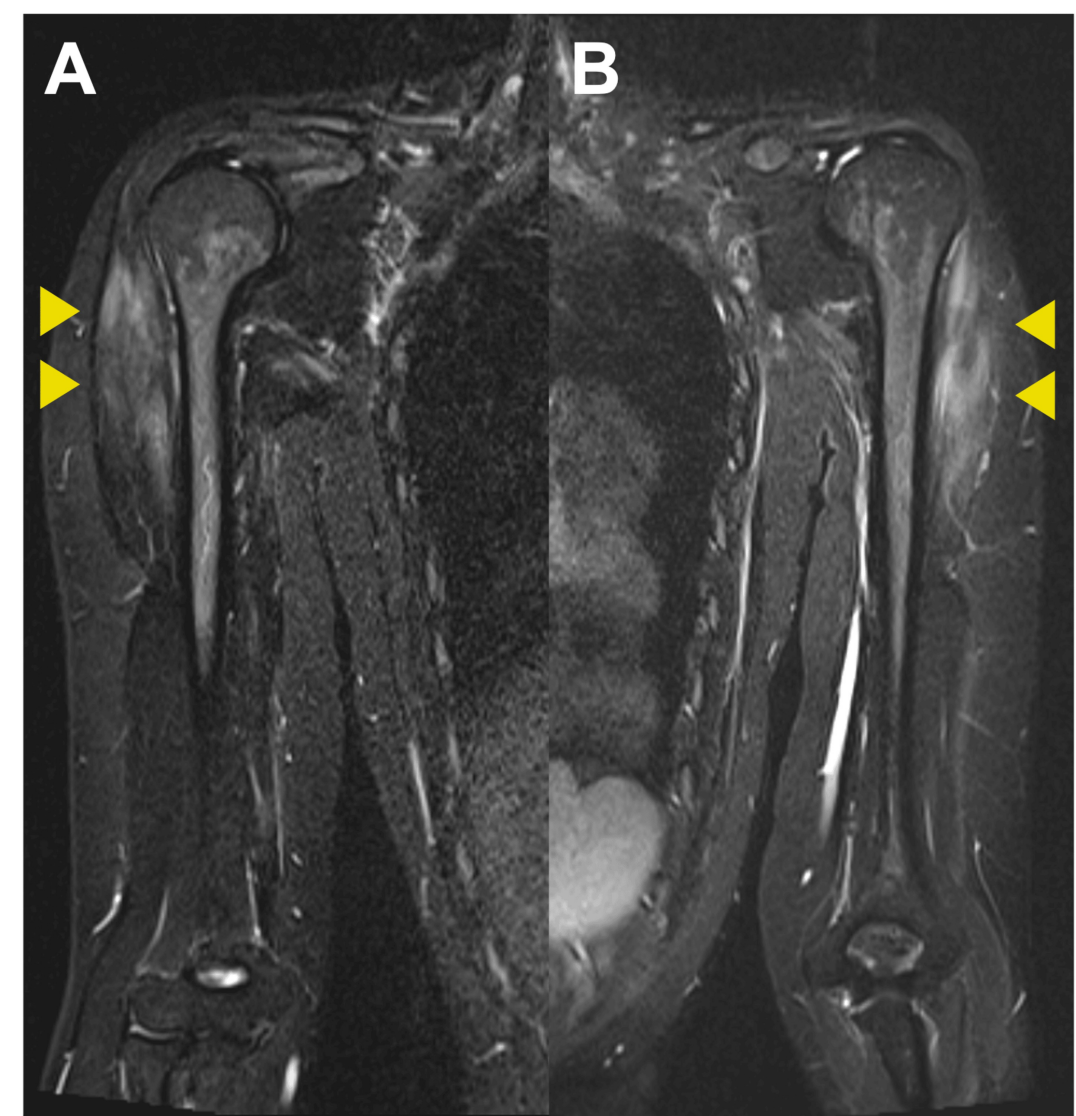
Non-contrast MRI of the patient’s arms. Short tau inversion recovery demonstrated high-intensity lesions in the right (A) and left (B) arm muscles (arrows).

Non-contrast MRI of the patient’s arms at this visit demonstrated findings suggestive of inflammatory myopathy (Figures 3A, 3B).

**Figure 4:**
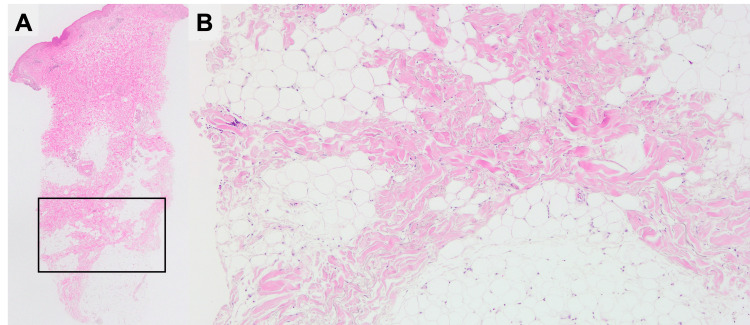
A skin biopsy of the erythema on the left thigh. The sclerotic lesions within subcutaneous adipose tissue. Original magnification ×20 (A), ×100 (B).

A muscle biopsy of the left biceps brachii revealed no degenerating or regenerating fibers, fibrosis, or vascular abnormalities were observed. Immunohistochemical staining showed mildly aberrant expression of MHC class I antigen in nearly all the fibers, while myxovirus resistance protein A protein expression was absent. A skin biopsy of the erythema on her left thigh revealed fibrotic lesions within the subcutaneous adipose tissue, which were consistent with healed panniculitis (Figures 4A, 4B).

**Figure 5:**
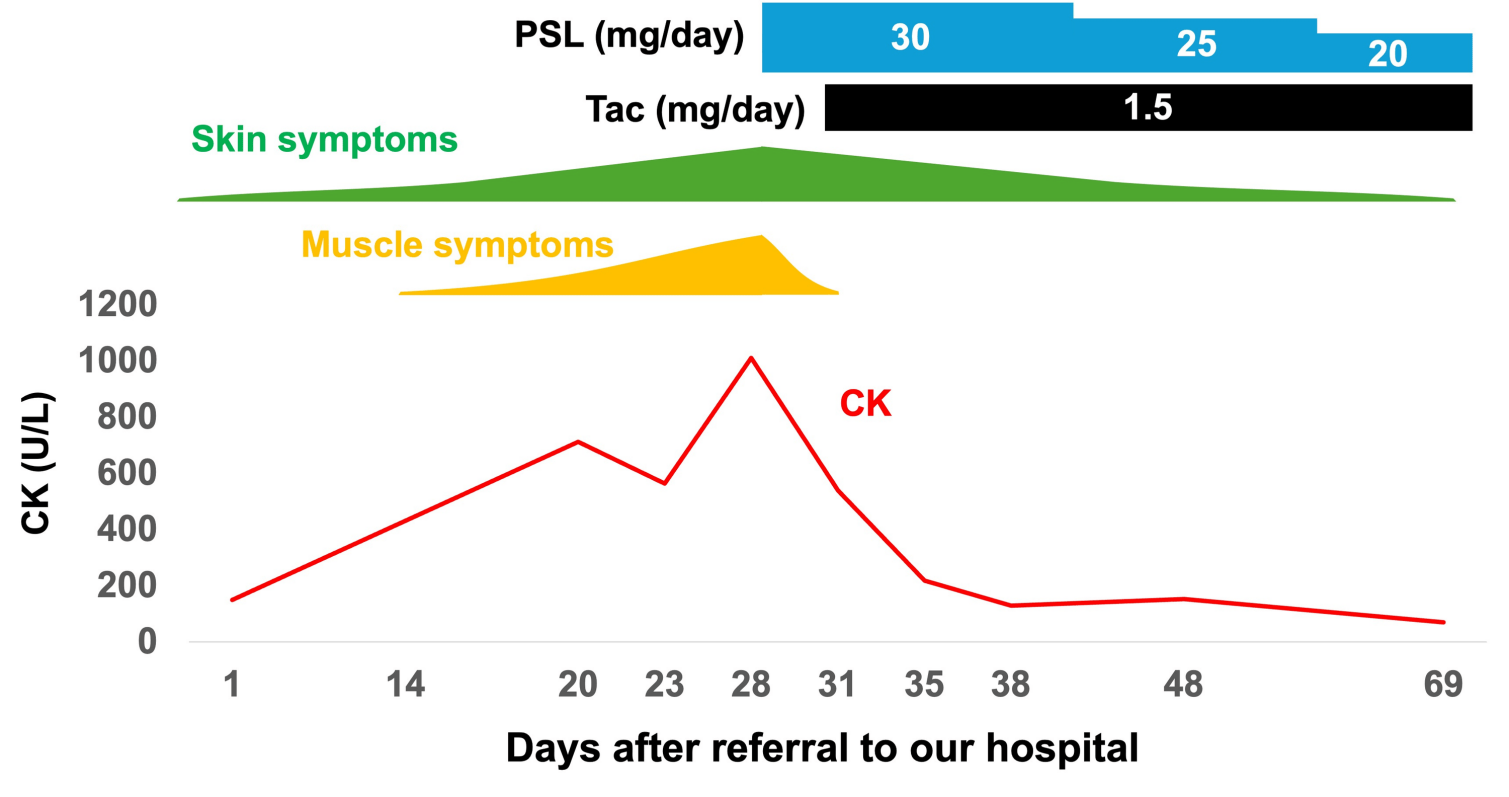
Clinical course of the symptoms, creatinine kinase, and treatment. CK, creatine kinase; PSL, prednisolone; Tac, tacrolimus

Non-contrast whole-body computed tomography found nothing to indicate malignancy or interstitial lung disease. Based on these results, anti-NXP-2-positive DM complicated by panniculitis was diagnosed, and PSL 30 mg/day (0.6 mg/kg/day) and tacrolimus 1.5 mg/day were administered as treatment. Subsequently, the erythema on the left thigh improved, leaving an area of skin pigmentation (Figures 1C, 1D). Her upper limb weakness also improved, and the serum CK value normalized. Figure 5 shows the details of the patient’s clinical course.

## Discussion

DM is a form of idiopathic inflammatory myopathy and has the hallmark symptoms of specific cutaneous lesions, including heliotrope rash, Gottron’s papules, and Gottron’s sign [7]. Panniculitis is a relatively rare skin manifestation of adult and JDM [1,8], and there are few reports of it as the initial symptom of DM [4]. Some studies have found an association between MSA, especially the anti-MDA5 antibody, and panniculitis [2,9,10]. DM-related panniculitis commonly presents with tender erythema in the extremities and the buttocks [8,11]. DM and panniculitis typically develop concurrently; however, in 17.5% of cases, panniculitis precedes myositis [2], sometimes for as long as one year [8]. In general, panniculitis is pathologically classified into lobular or septal type [12]. Lobular panniculitis, characterized by T-lymphocyte infiltration, necrotic adipocytes, and lymphocytic vasculitis, is the chief histological finding of DM-related panniculitis [13]. Additionally, in the late phase of DM-associated panniculitis, calcinosis, hyaline necrosis of adipose tissues, and subcutaneous sclerotic lesions may be observed [13,14]. In some cases, panniculitis is complicated by subcutaneous calcinosis or may heal, only to leave a lipoatrophic area [8]. In the present case, the erythema on the left thigh nine years ago resolved, leaving a lipoatrophic area. Although no skin biopsy of this lesion was performed at the time, the clinical course of this lesion was consistent with that of panniculitis. At the current presentation, a skin biopsy of the erythema on the left thigh revealed healed panniculitis. Based on these observations, anti-NXP-2 positive DM-related panniculitis was diagnosed.

Early diagnosis and treatment of DM-associated panniculitis are crucial for cosmetic reasons. As described above, panniculitis can leave areas of lipoatrophy which are characterized by the selective loss of adipose tissue [15] and may cause patients significant concerns about their appearance [16]. A systematic review reported that DM-associated panniculitis was more common in young women [2]. A previous study reported lipoatrophy as a complication in 18.6% (8/43) of cases of DM-associated panniculitis and a duration of 0-10 years (median 2.5 years) from the diagnosis of myositis to the development of lipoatrophy [8]. A retrospective study reported that 9.7% of 490 patients with JDM experienced the complication of lipoatrophy, which was associated with a longer duration of active DM [17]. As there is no cure for lipoatrophy [18], treating DM-associated panniculitis in its early stages is crucial for its prevention. In the present case, the DM relapse was recognized quickly, enabling early treatment.

## Conclusions

Physicians should recognize panniculitis as an important complication of DM. DM-associated panniculitis can leave areas of lipoatrophy, which may cause patients significant, cosmetic concerns. There is no cure for lipoatrophy, for the prevention of which the early treatment of panniculitis is crucial. Physicians should also be aware that DM, which is in remission, as in the present patient, may relapse with panniculitis in the absence of any accompanying muscle symptoms.

## References

[1] Hansen CB, Callen JP (2010). Connective tissue panniculitis: lupus panniculitis, dermatomyositis, morphea/scleroderma. Dermatol Ther.

[2] Ho JD, McKenzie T (2025). Panniculitis in dermatomyositis: a systematic review of the clinicopathologic features. JAAD Int.

[3] Fusade T, Belanyi P, Joly P, Thomine E, Mihout MF, Lauret P (1993). Subcutaneous changes in dermatomyositis. Br J Dermatol.

[4] Chao YY, Yang LJ (2000). Dermatomyositis presenting as panniculitis. Int J Dermatol.

[5] Papadopoulou C, Chew C, Wilkinson MG, McCann L, Wedderburn LR (2023). Juvenile idiopathic inflammatory myositis: an update on pathophysiology and clinical care. Nat Rev Rheumatol.

[6] Li S, Sun C, Zhang L (2022). Clinical heterogeneity of patients with antinuclear matrix protein 2 antibody-positive myositis: a retrospective cohort study in China. J Rheumatol.

[7] DeWane ME, Waldman R, Lu J (2020). Dermatomyositis: clinical features and pathogenesis. J Am Acad Dermatol.

[8] Suzon B, Goulabchand R, Louis-Sidney F (2023). Subcutaneous tissue involvement in idiopathic inflammatory myopathies: systematic literature review including three new cases and hypothetical mechanisms. Autoimmun Rev.

[9] Fiorentino D, Chung L, Zwerner J, Rosen A, Casciola-Rosen L (2011). The mucocutaneous and systemic phenotype of dermatomyositis patients with antibodies to MDA5 (CADM-140): a retrospective study. J Am Acad Dermatol.

[10] Labrador-Horrillo M, Martinez MA, Selva-O'Callaghan A, Trallero-Araguas E, Balada E, Vilardell-Tarres M, Juárez C (2014). Anti-MDA5 antibodies in a large Mediterranean population of adults with dermatomyositis. J Immunol Res.

[11] Hemmi S, Kushida R, Nishimura H, Murakami T, Sunada Y (2010). Magnetic resonance imaging diagnosis of panniculitis in dermatomyositis. Muscle Nerve.

[12] Requena L, Yus ES (2001). Panniculitis. Part I. Mostly septal panniculitis. J Am Acad Dermatol.

[13] Santos-Briz A, Calle A, Linos K (2018). Dermatomyositis panniculitis: a clinicopathological and immunohistochemical study of 18 cases. J Eur Acad Dermatol Venereol.

[14] Samanta J, Naidu GS, Sood R, Nada R, Sharma A, Jain S (2022). Calcinosis cutis with amyopathic dermatomyositis. QJM.

[15] Garg A (2004). Acquired and inherited lipodystrophies. N Engl J Med.

[16] Torrelo A, Noguera-Morel L, Hernández-Martín A (2017). Recurrent lipoatrophic panniculitis of children. J Eur Acad Dermatol Venereol.

[17] Ravelli A, Trail L, Ferrari C (2010). Long-term outcome and prognostic factors of juvenile dermatomyositis: a multinational, multicenter study of 490 patients. Arthritis Care Res (Hoboken).

[18] Brown RJ, Araujo-Vilar D, Cheung PT (2016). The diagnosis and management of lipodystrophy syndromes: a multi-Society practice guideline. J Clin Endocrinol Metab.

